# Knowledge, Attitudes and Perceptions of COVID-19 Vaccination among Healthcare Workers of an Inner-City Hospital in New York

**DOI:** 10.3390/vaccines9050516

**Published:** 2021-05-17

**Authors:** Federico Ciardi, Vidya Menon, Jamie L. Jensen, Masood A Shariff, Anjana Pillai, Usha Venugopal, Moiz Kasubhai, Vihren Dimitrov, Balavenkatesh Kanna, Brian D. Poole

**Affiliations:** 1 Department of Medicine, NYC Health and Hospitals/Lincoln, The Bronx, NY 10451, USA; ciardif@nychhc.org (F.C.); shariffm@nychhc.org (M.A.S.); anjana.pillai@nychhc.org (A.P.); usha.venugopal@nychhc.org (U.V.); Moiz.Kasubhai@nychhc.org (M.K.); vihren.dimitrov@nychhc.org (V.D.); balavenkatesh.kanna@nychhc.org (B.K.); 2 Department of Biology, Brigham Young University, Provo, UT 84602, USA; jamie.jensen@byu.edu; 3 Department of Microbiology and Molecular Biology, Brigham Young University, Provo, UT 84602, USA; brian_poole@byu.edu

**Keywords:** COVID-19, vaccine hesitancy, healthcare workers, vaccine attitudes

## Abstract

**Introduction:** New York City is one of the areas most affected by the COVID-19 pandemic in the United States. Healthcare workers are among those at high risk of contracting the virus, and a vital source of information and trust in vaccines to the community. **Methods:** This study was conducted about attitudes towards COVID-19 vaccination among healthcare workers at a public hospital in New York City during the beginning of COVID-19 vaccination. 428 hospital employees responded. **Results:** Several factors were significantly associated with vaccine attitudes, including demographics such as gender (*p* = 0.002), age (*p* = 0.005), race (*p* < 0.001) and home location (*p* < 0.001), role within the hospital (*p* < 0.001), knowledge about the virus (*p* < 0.001) and confidence in and expectations about personal protective equipment and behaviors (*p* < 0.001). Structural equation modeling revealed that the most predictive factors were prior vaccine attitudes and concern with the speed of testing and approval of the vaccines (*p* < 0.001). Multivariate analysis reinforced these, while also identifying perceived personal risk as significant (*p* = 0.033). **Conclusions:** Several modifiable factors that reflect confidence in science, scientific knowledge, personal risk perception, experience and medical authority are correlated with vaccine attitudes, indicating that a holistic educational approach to improve trust in science is likely to be effective in long-term reduction in vaccine hesitancy.

## 1. Introduction

Since December 2019, when the SARS-CoV-2 novel coronavirus was first reported in Wuhan, China, the pandemic has infected more than 160 million people and claimed more than 3.3 million lives [1]. Beyond the disease itself, unprecedented social and economic hardship has unveiled across the globe due to this infection. Development of a vaccine against the virus is considered a pivotal moment in the efforts to curb disease spread and begin the resumption of normalcy in everyday life. As of 18 February 2021, at least seven different vaccines across three platforms have been distributed globally according to the World Health Organization (WHO) [2]. Despite this unprecedented scientific discovery, vaccine hesitancy is seen as a stumbling block towards achieving herd immunity in the battle to control this global pandemic.

In 2019, the WHO identified vaccine hesitancy as one of the top ten global health threats [3,4]. Misinformation, lack of trust in key industry players and poor communication with populations have been the drivers of this trend over the past 15 years. The Pew Research Center reported on December 3rd, 2020 that 39% of Americans would probably, or definitely, not get a vaccine against COVID-19 [5]. Gallup similarly reported that between 16–29 November, 37% of poll respondents would not receive a Food and Drug Administration (FDA)-approved vaccine at no cost [6]. A nationally representative longitudinal survey done from April to December 2020, showed a reduction in the likelihood of getting COVID-19 vaccination from 74% in early April to 56% in December, despite the positive press releases and information about the mRNA vaccine efficacy [7] These are alarming figures, considering that most estimates of the proportion of Americans that would need to be immune to achieve herd protection (otherwise, herd immunity) are between 60% and 80% [8,9].

Investigations into public attitudes during the pandemic have revealed details on vaccine and recipient features that are likely to influence eventual uptake. Kreps et al. reported that increased efficacy and duration of protection, with decreased incidence of major adverse effects and full FDA approval, appear to increase willingness of Americans to receive the vaccine against COVID-19 [10]. Other published surveys suggest recipient factors that decrease willingness to accept a vaccine include younger age, minority ethnic groups, not being a healthcare worker and lower individual perceived risk [11]. Of note, individuals in the UK with a pre-existing respiratory condition reported an 86% acceptance of a COVID-19 vaccine that was correlated with increased perception of personal morbidity [12]. The development of vaccines in record time, release under emergency use authorization (EUA) rather than full FDA approval, perceived political interference and superficial safety reviews, are important causes for skepticism [13]. With respect to healthcare workers specifically, a survey conducted on patient-facing members of the Association of Hong Kong Nursing Staff in the early months of the pandemic reported just 40% acceptance of a COVID-19 vaccine [14]. More recently a large, multicenter survey of healthcare workers conducted between October and November 2020 in midwest and southwest states showed striking uncertainty in the months leading up to the release of preliminary safety data, with only 36% of respondents immediately accepting, and 56% undecided [15]. Importantly, prior reports in the healthcare setting have failed to include an adequately diverse sample population, which has resulted so far in limited data on between ethnic group analyses. This is concerning as data from diverse, public surveys [7,16] identify this as a principal discriminator of vaccine acceptance and hesitance.

During the first surge in the spring of 2020, the New York City (NYC) public health care system expanded capacity swiftly across all acute care hospitals to respond to the unprecedented demand for patient care in response to the COVID-19 pandemic. The South Bronx was the epicenter of the pandemic during the first surge and continues to have high community positivity rates and fatalities [17]. NYC hospitals were among the first in the nation to receive vaccines as part of the public health strategy to immunize high risk health care workers and high-risk individuals. Our study sampled a cross-section of healthcare workers from this area on their understanding of, and attitudes regarding, COVID-19 vaccination with an aim to provide a deeper insight into the factors that need to be addressed to reduce vaccine hesitancy. The sampled population not only serves a diverse and low income community but is itself predominantly made up of ethnic minorities that specifically reflect the most vaccine hesitant and vulnerable groups. To achieve herd protection and defeat Covid-19 it is necessary to understand, educate and include this vaccine-resistant cohort and, in doing so, reduce the healthcare disparity across the United States.

## 2. Materials and Methods

### 2.1. Settings and Survey Participants

Healthcare workers from a NYC public hospital in the South Bronx participated in a cross-sectional study by completing an online survey developed to understand their knowledge, attitude and perceptions about the COVID-19 vaccination. They were provided with an anonymous link to the survey, which was administered by Qualtrics (Provo, UT). Upon selecting the survey link, the participants took the survey over the internet. The survey period was open from 10 December 2020 to 5 January 2021. Only complete responses were recorded and counted. Surveys completed by respondents with the same IP address were excluded as they were considered as an overlapping response. No cap was set on participation, so all who were invited were able to participate. Institutional Review Board (IRB) approval was obtained (IRB # 20-043). A total of 428 responses were recorded, which amounts to a response rate of approximately 10% of all healthcare workers within the institution.

### 2.2. Survey Description

The survey assessed the participants’ knowledge about the impact of COVID-19 infection, vaccinations in general and COVID-19 vaccination. Knowledge of COVID-19 was measured using a scale including the questions “Vaccines against pneumonia can protect against COVID-19.,” “Certain antibiotics can protect against COVID-19,” “On average it takes 5 to 6 days from when someone is infected with COVID-19 for symptoms to show; however it can take up to 14 days,” “Regularly rinsing your nose with saline can help prevent infection with COVID-19,” “Once you contract COVID-19, the virus can never be eliminated from your body,” “Symptoms of COVID-19 can include sore throat, diarrhea, and conjunctivitis (eye infection)” and “Most people who contract COVID-19 will recover from it.” 

Sociodemographic characteristics including age, gender, race, marital status, number of children, level of education, primary role, location of work and primary source of COVID-19 related information were obtained from all participants. Questions in the survey sought to understand the intention and behavior of participants regarding the COVID-19 vaccination and the factors influencing their decisions towards acceptance, hesitancy or denial of vaccination. Based on previously published data that personal exposure to individuals suffering from COVID-19 would decrease vaccine hesitancy, respondents were asked to identify the most severe outcome of infection among people they knew personally, as well as the closeness of the relationship [4]. The participants’ attitudes and practices towards the effectiveness of personal protective equipment (PPE), such as masks, and practices like social distancing, were also measured. Participants were also asked whether, after vaccination, they could stop doing the following: wearing masks, social distancing in public, social distancing at work, wearing PPE at work and washing hands in between patients, with responses requested on a 5-point Likert scale. The entire survey is available in the Supplementary Section (Supplementary Data 1).

A latent variable structure was set up for several items hypothesized to predict a person’s intention to receive the COVID-19 vaccine. These latent variables were as follows: (1) the respondent’s perception of personal risk concerning COVID-19; (2) attitudes and behaviors toward vaccines in general; (3) concerns about the country in which the COVID-19 vaccine was developed and (4) concerns about the speed at which the vaccine has been tested and approved. Further details of each latent variable are described in the Results section. 

Intention regarding the COVID-19 vaccine was measured using the items “In the next 30 days (I plan to vaccinate myself or I do not plan to vaccinate myself),” “In the next six months (I plan to vaccinate myself or I do not plan to vaccinate myself),” “If the COVID-19 vaccine would need to be administered yearly (similar to the flu shot), would you receive repeated vaccinations?,” “If a vaccine for COVID-19 was made available and you were told it would protect 3/4 of those who received it, how likely would you be to be vaccinated?” and “If a vaccine for COVID-19 was made available and you were told it would protect 90% of those who received it, how likely would you be to be vaccinated?” These measures were combined into the COVID-19 Vaccine Score. The vaccine score was used for the Structural Equation model and for univariate analysis with continuous variables such as confidence in PPE. The complete survey is available in the Supplementary Materials (Table S1).

### 2.3. Model Fit and Structural Equation Modeling

We used confirmatory factor analysis (CFA) on the measurement portion of our model, and structural equation modeling (SEM) on the structural part of our model to determine relationships between multiple variables, utilizing the Mplus software, version 8 (Muthen and Muthen, 1998–2010). CFA was performed on all latent variables in the model, including intention to receive the COVID-19 vaccine, with a request for modification indices. Items were removed until fit indices (root mean square error of approximation (RMSEA), comparative fit index (CFI), Tucker-Lewis index (TLI), standardized root mean square residual (SRMR), and Chi square (χ^2^)) were acceptable. A hypothesized structural model was determined and SEM was performed using our latent variables; highest level of education and political leanings were also included as covariates. We used the full information maximum likelihood method to deal with missing data. The final model was selected based on fit statistics, as described in Results. 

### 2.4. Regression

To follow up on the structural modeling, we performed a multiple regression analysis. To create each predictor, we summed responses for each item to obtain a composite score for each latent variable (some items were reverse coded so that they were all coded in the same direction). The data met most assumptions (independence, linearity and no multicollinearity). However, due to the mostly positive attitudes and, therefore, positive skew in our outcome variable attitudes toward the COVID-19 vaccine, our data showed heteroscedasticity. Thus, we used a Generalized Linear Model utilizing a gamma probability distribution. 

### 2.5. Univariate Analysis

When responses were categorical, such as location of residence and role within the hospital, these were measured by chi-square analysis using the question “in the next 30 days…” with possible responses “I plan to vaccinate myself” or “I do not plan to vaccinate myself”.

The response was used as it was the most direct answer to the question of current intention towards the vaccine. When variables were continuous, such as those using a 5-point Likert scale, the COVID-19 vaccine attitude score used in the SEM model, which included 30 day and 6-month intention, contributions of vaccine effectiveness, and the contribute of the potential need for booster immunizations, was used for correlation. A higher score on the COVID-19 attitude scale means higher receptiveness to COVID-19 vaccination. Correlations were examined using Pearson r values.

## 3. Results

### 3.1. Characteristics of the Study Cohort

Table 1 describes the baseline characteristics of the cohort. A majority of the respondents were women (65%) and the age range was between 26 and 45 years (54%). Latinx (29%) and White (24%) race were predominant, followed by Asian (20%) and African Americans (19%). Of the respondents, 51% were married and 41% were single. There was a high level of education among those surveyed: 34% had a doctoral degree and 17% had a master’s degree, whereas less than 4% had no college education, which reflects the underlying population of hospital employees. Doctors (28.5%) and nurses (22%) made up the majority of respondents. Individuals recorded working mostly in ambulatory care (20%), the emergency department (19%) and medical/surgical inpatient units (19%), with a lower proportion working in administrative offices (11%) or the operating department (5%). Finally, 35% of respondents lived in the Bronx, 19% in Manhattan, 11% in Queens and 13% outside of New York City.

### 3.2. Univariate and Descriptive Analysis

Among the subjects who took the survey, 64% were planning on being vaccinated in 30 days. Significantly more (73%) were planning on being vaccinated in the next six months (*p* = 0.002).

#### 3.2.1. Demographic Factors

**Figure 1:**
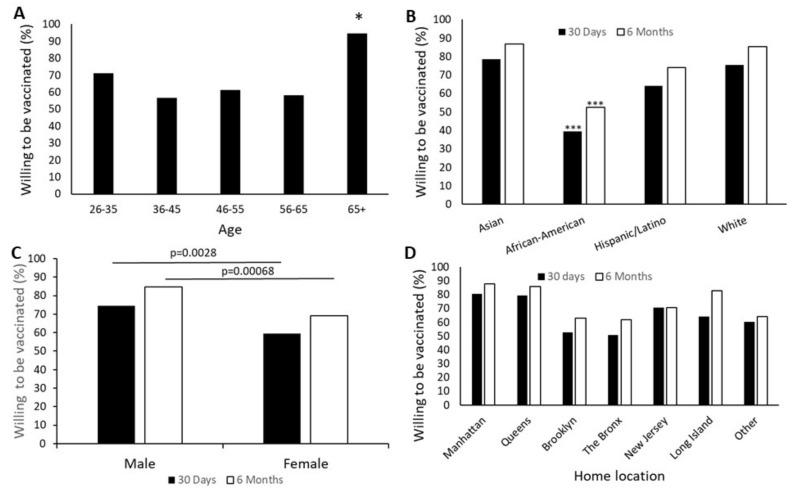
Demographic influences on vaccine acceptance. Willingness to be vaccinated was evaluated using the questions “In the next 30 days…” or “In the next 6 months…” with responses being “I plan to vaccinate myself” or “I will not vaccinate myself.” (**A**) Significantly more subjects over the age of 65 were planning to be vaccinated within 30 days than any other group (Overall, χ2 = 14.65, *p* = 0.005, comparison of 65+ to each other group (*) *p* < 0.030). This difference disappeared when the time frame was extended to six months. (**B**) African American participants were significantly less likely to plan on vaccination than any other ethnic group within either 30 days or six months (*** *p* < 0.0003), (**C**) Women were less likely than men to accept vaccination within either time frame (*p* < 0.001). (**D**) Location of a person’s home was statistically meaningful in COVID-19 vaccination intention during both time frames (χ^2^ > 26, *p* < 0.0003). *n* = 428.

Age, gender and race all showed significant associations with willingness to be vaccinated. The oldest group, more than 65 years, were the most likely to accept vaccination within the next 30 days, with 95% acceptance. The next most willing subgroup was the youngest cohort 26–35 years, with 71% acceptance. The age group least willing to accept vaccination was 36–45 years, with only 57% planning to be vaccinated in 30 days from the time of the study (Figure 1). These patterns persisted when the time frame for vaccination was extended to six months, with the exception that the group with the least intention to be vaccinated during the next six months was 56–65-year-old. However, these differences were not significant in the 6-month group. 

Willingness to be vaccinated also significantly depended on race (χ^2^ = 1.24 − 10^−7^, *p* < 0.001. Asian respondents had the highest willingness to accept vaccination within 30 days (79%), while African American subjects had significantly lower acceptance than every other race/ethnicity (40%, *p* < 0.001). Hispanic individuals were also significantly less willing to accept vaccination than Asian respondents (*p* = 0.027). Over a six-month timeframe, these patterns remained the same, with African American respondents least planning to receive the vaccine (*p* < 0.002) (Figure 1). There were too few Native American respondents for statistical analysis (*n* = 3) but all three were unwilling to be vaccinated in the next 30 days.

Gender was a significant determinant of immediate vaccine acceptance. Although 75% of male respondents were planning on being vaccinated within 30 days, only 60% of female respondents (χ^2^ = 8.99, *p* = 0.002) were (Figure 1). Analysis of attitudes about vaccination was done using grouping by age to see if concern about effects of the COVID-19 vaccine on reproductive ability among the women of childbearing age group explained this difference and did not suggest that women of childbearing age differed from the overall female population. Male respondents remained more likely to plan on being vaccinated in the next six months than female respondents (*p* < 0.001).

Where the respondent lived significantly impacted their willingness to be vaccinated within 30 days (χ^2^ = 28.63, *p* < 0.001). Individuals from The Bronx showed the lowest enthusiasm for vaccination, with only 51% indicating an intent to be vaccinated. Participants who lived in Manhattan had the highest level of enthusiasm for vaccination at 81%. (Figure 1).

#### 3.2.2. Participant Role and Attitude towards COVID-19 Vaccination

**Figure 2:**
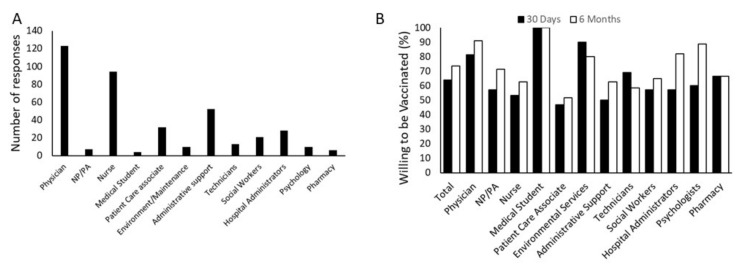
Influence of hospital role on vaccine acceptance. (**A**) Survey responses were unevenly distributed among hospital employees, with an abundance of physicians and nurses. However, multiple roles in all aspects of hospital employment are represented. (NP/PA-Nurse practitioner or Physician’s assistant) (**B**) Willingness to be vaccinated was evaluated by the response to the questions “In the next 30 days…” or “In the next 6 months…” with responses being “I plan to vaccinate myself” or “I will not vaccinate myself.” The respondent’s role in the hospital significantly affects their plan to be vaccinated within both 30 days (χ2 = 35.69, *p* < 0.001) and six months (χ2 = 40.22, *p* < 0.001) *N* = 426.

The intent to be vaccinated in 30 days was significantly dependent on the role of the respondent in the health care system (χ^2^ 35.69, *p* < 0.001). Intent ranged from 47% among the patient care associates to a high of 100% among the medical students, although only five medical students were included in the survey (Figure 2). When the time frame was extended to six months, the same patterns remained (χ^2^ 40.22, *p* < 0.001), although intent to be vaccinated was slightly higher for most groups.

#### 3.2.3. PPE, Preventive Practices and Attitude towards COVID-19 Vaccination

**Figure 3:**
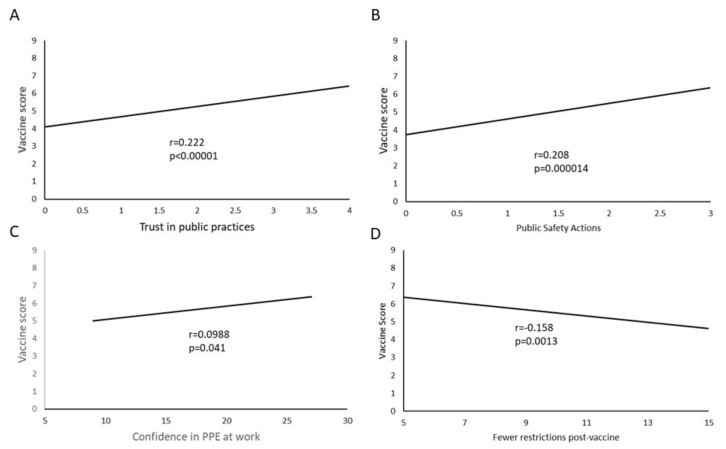
Personal Protective equipment (PPE) practices correlate with vaccine attitudes. (**A**) Participants with high trust in PPE and behavioral actions in public also have more intention to be vaccinated as measured by the COVID-19 vaccine attitude score. (**B**) Participants who use masks and behavioral modifications in public also have higher intention to be vaccinated. (**C**) Confidence in PPE and behavioral modification to keep the participants and colleagues safe from infection in the hospital is mildly correlated with increased intent to vaccinate. (**D**) Intent to vaccinate is correlated with the expectation that protective equipment and practices will need to be continued even after vaccination. Sample size for each was *N* = 428. Only trendlines are shown for each of the dot plots to decrease noise.

A strong correlation existed between a belief that PPE and public health measures are beneficial and a high COVID-19 vaccine attitude score (*r* = 0.222, *p* < 0.001). Similarly, there was a strong correlation between the reported action of using these measures in public and a high COVID-19 vaccine attitude score (*r* = 0.208, *p* < 0.001) (Figure 3).

To assess how the respondents felt about how effective protective measures are in the hospital, a battery of nine items were assessed and added together to give a “protective measures” score. The items were responses to the statement: “Please indicate how confident you are that each of the following measures will protect you and your colleagues from contracting COVID-19 at work”. The individual measures were: N95 masks, correct use of protective eyewear, correct use of gloves, correct use of aprons/gowns, patient isolation, negative pressure rooms, rapid testing for patients upon admission, mobile daily staff screening and cleaning and disinfecting surfaces and rooms. This was then compared to the COVID-19 vaccine attitude score. A small positive correlation was found between the two (r = 0.099, *p* = 0.040) (Figure 3).

With respect to the ability to resume normal activity and dispense with protective practices after vaccination, the responses were summed and scored so that a lower number corresponded to less expected relaxation of protective standards and practices. There was a negative correlation between expectation of relaxed protective practices and COVID-19 vaccine score (r = −0.158, *p* = 0.001) (Figure 3), suggesting that individuals with a high intention to be vaccinated still expected the need to use PPE and protective practices after vaccination. There was no significant difference in intent to be vaccinated based on presence or absence of COVID-19 antibodies prior to vaccination. 

#### 3.2.4. Information and Attitude towards COVID-19 Vaccination

**Figure 4:**
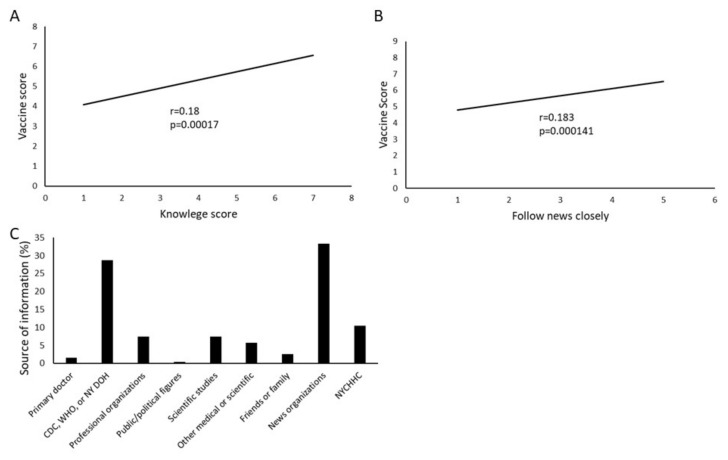
COVID-19 knowledge and vaccine attitudes. (**A**) Scores on the COVD-19 knowledge section of the survey strongly correlate with the COVID vaccine score, indicating a higher intent to vaccinate. (**B**) Similarly, closely following news of COVID-19 correlated with willingness to be vaccinated. (**C**) Sources of information varied widely, with news organizations the most common, followed by government sources. (NYCHHC-New York City Health and Hospitals). *N* = 428. Only trendlines are shown for each of the dot plots to clearly show the correlations.

A strong correlation was seen between participants’ knowledge about COVID-19 infection and positive attitude towards receiving COVID-19 vaccination (r = 0.18, *p* < 0.001) (Figure 4). Similarly, subjects who followed news more closely had an increased vaccine score (r = 0.183, *p* < 0.001). The most common source of information sought by the survey participants for COVID-19 related knowledge was news organizations, followed by government sources (CDC, WHO, or the New York City Department of Health) (Figure 4).

#### 3.2.5. Personal Experience and Attitude towards COVID-19 Vaccination

**Figure 5:**
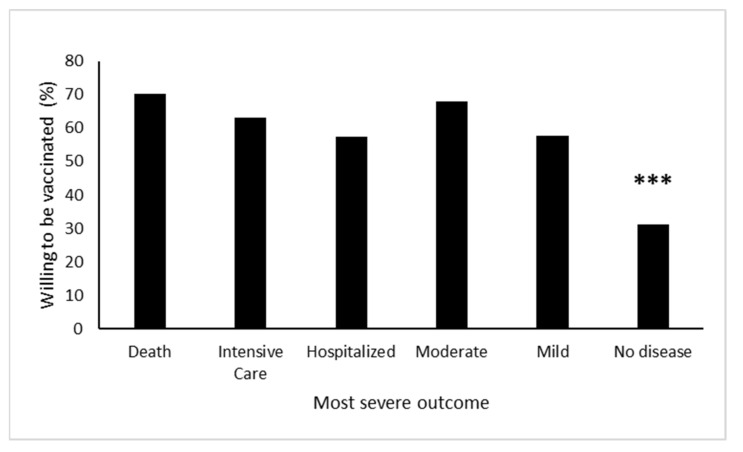
Experience with COVID-19 is associated with intent to vaccinate. Respondents who had personal experience with someone with COVID-19 disease were significantly more likely to report being willing to be vaccinated in the next 30 days (*** χ2 = 19.26, *p* < 0.001). No significant differences were found between any groups who had personal experience with disease patients. The respondents were asked to list the most severe outcome if they knew more than one person with COVID-19.

Personal experience with someone with COVID-19 infection significantly increased intent to be vaccinated in the next 30 days (χ^2^ = 19.26, *p* < 0.001). Interestingly, however, the severity of illness was not significant in influencing their decision about vaccination. Subjects with any type of personal experience with COVID-19 were significantly more likely to say they would be vaccinated than those without. Similarly, the closeness of the contact who had COVID-19 did not have any effect on the intent to be vaccinated (Figure 5).

### 3.3. Modeling and Multivariate Analysis

#### 3.3.1. Statistical Results of CFA and SEM

CFA was used to confirm that each item appropriately measured our latent variables. Based on suggested modifications, ‘with’ statements were included in the model and some items were removed to improve fit. The items retained for each factor along with the fit statistics for each model are shown in Table 2. Factor loadings for each item were acceptable. All loadings were significant at *p* < 0.05. Our CFA confirmed that our instrument measured distinct and identifiable factors.

#### 3.3.2. Structural Equation Model (SEM)

**Figure 6:**
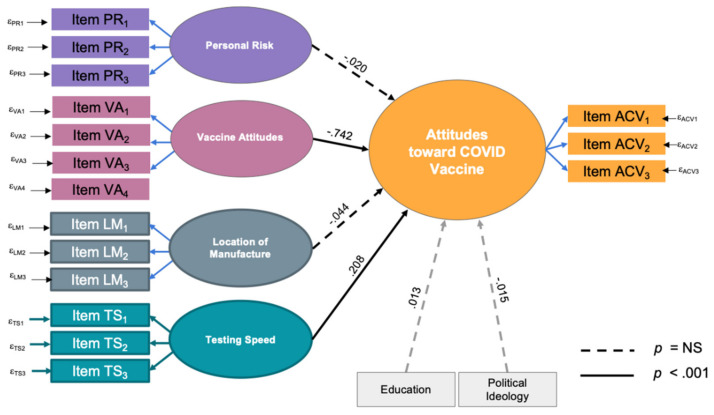
Structural equation model. Four variables, each represented by three survey items, were tested for model fit, and then analyzed for prediction of the output variable, attitudes toward COVID vaccination. Each rectangle represents a survey item as described in Table 2. Solid lines show statistically significant predictive ability while dashed lines show nonsignificant associations. Negative overall vaccine attitudes and a strong perception of too-rapid vaccine testing were predictive of worse attitudes towards receiving a COVID-19 vaccine. Education and political ideology were tested as covariates and did not show significant prediction of COVID-19 vaccine attitudes.

Structural equation modeling indicated two significant predictors of the likelihood of accepting the COVID-19 vaccine: general attitudes toward vaccines and concern with the speed of testing. The better an individual’s attitudes toward vaccines in general, the more likely they were to receive the COVID-19 vaccine. Additionally, the more concerned they are over the speed of research and FDA approval, the less likely they are to get the vaccine. A person’s sense of personal risk and worries about the location of manufacture had no significant predictive value on attitudes toward the COVID-19 vaccine. Interesting, contrary to popular belief, level of education and political leanings did not significantly influence attitudes toward the COVID-19 vaccine. The structural model showed a robust fit for the data as indicated by fit statistics and probability scores (RMSEA = 0.063, CFI = 0.910, TLI = 0.877, SRMR = 0.071, χ^2^ = 2258.67, *p* < 0.001). The model, with standardized estimates for relationships is shown in (Figure 6).

#### 3.3.3. Regression Analysis

A model including each summed variable was significant in predicting COVID-19 vaccine attitudes (χ^2^(6) = 196.63, *p* < 0.001). Significant predictors once again included attitudes toward vaccines in general (χ^2^(1) = 202.37, *p* < 0.001) and concerns with the speed of testing (χ^2^(1) = 7.30, *p* = 0.007). Additionally, a perception of personal risk was a small but significant predictor (χ^2^(1) = 4.57, *p* = 0.033). Regression coefficients and standard errors are shown in Table 3. 

## 4. Discussion

The tremendous success in getting the COVID-19 vaccine candidates from “bench to bedside” at a remarkable speed to meet the public health need is a testament to modern scientific technology. However, it is equally critical to ensure the vaccine is administered equitably to the entire population to achieve desired herd immunity. While all healthcare institutions rushed to provide the COVID-19 vaccines to their staff, significant disparity was observed in the uptake of the vaccinations between the private and public institutions in NYC [18]. Our cross-sectional survey, administered just before and during the initial period of the vaccine rollout, provides an understanding of the challenges which must be overcome to improve COVID-19 vaccination uptake among healthcare workers in our NYC public hospital. While almost 64% of respondents were willing to be vaccinated within 30 days, and a further 10% of respondents were willing after six months, 26% reported being unwilling even after six months of the initial rollout. Though the recorded vaccine acceptance rates are similar to national and international studies published prior to the initial remarkable vaccine efficacy and safety data, it is significant to recognize that respondents still have major concerns regarding the vaccination, in spite of the tremendous burden of patients hospitalized with COVID-19 in New York earlier in the pandemic [1].

While vaccines are known to be successful public health measures, an increasing number of people believe vaccines are neither safe nor necessary [19]. This behavior is determined by issues of confidence or trust in the vaccine or provider, perceived lack of need or value for the vaccine and issues with access to the vaccine [20]. Two significant predictors of likelihood of vaccine acceptance in our study participants were general attitudes towards vaccination and concern about the speed of research, including testing in the population as well as FDA approval. The expedited development and novelty of the COVID-19 vaccines combined with inadequate public health and political messaging, have undermined the confidence of the population already reeling under the effects of a grueling pandemic in the background of harmful political rhetoric with an excess of misinformation, disinformation and conspiracy theories.

Healthcare workers include a variety of occupations with varying levels of education. Several aspects of the study touch on the importance of education and the ability to recognize correct information about the COVID-19 vaccine. First, we correlated vaccine acceptance with role at the hospital. This is a reasonable proxy for education, since there is something of an educational hierarchy within the hospital roles. Interestingly, although vaccine acceptance was dependent on hospital role, it was not strictly what would be expected based on education level; for example, nurses, who are highly educated and clinically trained, were less accepting of vaccination than those in environmental services.

Overall education level was also not found to be predictive when using the modeling or multivariate analyses (Table 3). However, correct knowledge of the virus as measured by the knowledge score was significantly associated with pro-vaccine attitudes (Figure 4). Therefore, it is likely that although education does have some effect on vaccine attitudes, the ability to find correct information is more important than a participant’s formal education level.

In the months following administration of this survey, vaccine uptake at this facility approached 70–75%, as anticipated by the combination of respondents willing to be vaccinated at 30 days and six months combined. We hypothesize that the smaller, initially hesitant, group who were not vaccinated immediately always intended to get vaccinated eventually but wanted to observe the experience of vaccination in their colleagues. Of greater concern is the remaining 25–30% whose opinions against vaccination appear to become more entrenched with time. The results presented herein help characterize this group in the healthcare worker setting and should be used to focus education and communication efforts in our industry to achieve the greatest vaccine uptake in vulnerable minority populations. It is crucially important to note our results demonstrating that young, female, African American individuals (Figure 1) with low pre-existing vaccine attitudes (Table 2) who do not have experience with Covid-19 (Figure 5), represent our most vaccine-resistant respondents; a cohort known to have consistently poorer health outcomes across the United States. Accurate knowledge about COVID-19 and relationships with those who suffered COVID-19 were associated with higher vaccine acceptance. These findings are in line with earlier research [4] and indicate that education programs or trainings where healthcare workers are involved in discussions with people who have suffered severe disease outcomes may be helpful in encouraging vaccine uptake.

While those aged 65 and above in our cohort were most willing to be vaccinated, we note that only 43% of the 36–45-year-old participants intended to be vaccinated. Similar results have been reported from multiple studies, and our results suggest that younger individuals perceive risk of infection to be less than the risk posed by vaccination [10,21]. This result may be confounded by only 19 (4.44%) respondents in the 65 and above age group, though this is nationally representative according to the U.S. Bureau of Labor Statistics in 2020 [22]. Racial disparities were observed in vaccine acceptance with Asian respondents being most willing, and African American respondents being least agreeable. Though 66% of the survey participants were women, we observed a significant male predominance in willingness to be vaccinated. Most studies of gender predisposition to COVID-19 vaccine acceptance show trends with women being more vaccine-hesitant, and more concerned about vaccine safety [10,23]. It has been hypothesized that the difference between male and female willingness to be vaccinated may reflect concerns about the unstudied effect on fertility, pregnancy and breastfeeding; a point addressed recently by Adhikari et al. in *JAMA* [24]. However, a subgroup analysis of our sample population (not shown here) between women in different age groups did not demonstrate a significant difference between those of childbearing age and others, suggesting that this may not, in fact, be an important factor in female health care workers’ decision. 

In our cohort, the area of residence also seemed to have an impact on vaccine attitude, with health care workers who lived in Manhattan being more willing than those from the Bronx. This is confirmed by the NYC health department, with data suggesting zip codes in Manhattan and Staten Island show higher vaccination rates than those in the South Bronx, central Queens or central Brooklyn [25].

Nurses and patient care associates were among those with the least intent to be vaccinated in comparison to medical students and physicians who were the highest. A survey of thirteen thousand nurses conducted by the American Nurses Foundation and published in October 2020, suggested only 34% of nurses were willing to be vaccinated with the COVID-19 vaccine, while 36% refused and 31% were unsure [26]. Similar trends of suboptimal COVID-19 vaccination uptake have been reported from a survey from Hong Kong, including 1205 nurses [27].

Subjects who believed and complied with effective PPE practices had a positive COVID-19 vaccine attitude and they believed in the need to continue to use PPE even after completion of vaccination. The finding that the expectation for continued PPE and social distancing measures correlated with increased vaccine acceptance may be counterintuitive because it may suggest that those who are more accepting of the vaccine have less confidence in its utility. A more likely explanation, however, is that people who are committed to community health, and have a greater understanding of the impact of protective measures, are also more likely to be vaccinated. These people may be more community-focused than oriented on how their own lives will change. Compliance with social distancing and PPE use is conformant with perception of a high-level threat due to COVID-19 among the population and is noted in an area where the impact of COVID-19 has been greater [28]. Previous exposure to COVID-19 infection, or presence of COVID-19 antibodies, did not have any bearing on our health care workers decision to be vaccinated. We observed a strong correlation between the subject’s knowledge about COVID-19 infection and personal experience with someone with COVID-19 with a positive attitude towards vaccination. Not surprisingly this group sought information about the pandemic by following the news and government sources, such as the CDC and the New York State Department of Health, while political affiliations or country of manufacture of the vaccine did not impact decision for vaccination.

### Limitations

The study has several limitations. First, only about 10% of all the healthcare workers in the surveyed institution participated; however the model fit statistics were robust. The suboptimal response rate may introduce significant selection bias into the results; however, distribution of the survey was circulated uniformly to all employees both in print and electronically. Consequently, generalizability of the study may also be limited as this was a single-center study with a moderate sample size. The survey was long and detailed, which allowed us to include many variables in our analysis and provide robust, granular insights into the knowledge, attitudes and perceptions of our study population. Second, timing of the survey distribution coincided with vaccine rollout that likely impacted the responses. The five weeks surrounding vaccine deployment were a highly dynamic and changing landscape during which respondents were overwhelmed with information, disinformation, and emotion, possibly leading to reactionary opinions expressed in their answers.

## 5. Conclusions

Vaccine acceptance in our cohort was depended on general attitudes towards vaccination and concern about the speed of research, including testing in the population, as well as FDA approval. Vaccine acceptance was also strongly associated with acceptance of other methods or COVID-19 control. Regrettably, perception and distrust of the medical research establishment apparently extends to healthcare workers, especially from minority communities, and may hinder public efforts to curb the spread of COVID-19. Notably the vaccine hesitancy seen among nurses calls for timely efforts to address the barriers and improve attitude and perception towards COVID-19 vaccination.

## Figures and Tables

**Table 1:** Participant characteristics.

Data	Number	Percentage of Total Responses
Age (*n* = 428)		
18–25	4	0.93%
26–35	125	29.21%
36–45	106	24.77%
46–55	88	20.56%
55–65	86	20.09%
>65	19	4.44%
Gender (*n* = 428)		
Male	137	32.01%
Female	279	65.19%
Prefer to self-describe	3	0.70%
Prefer not to answer	9	2.10%
Race (*n* = 428)		
White	102	23.83%
Latinx	123	28.74%
African American	82	19.16%
Asian	84	19.63%
Native American	3	0.70%
Native Hawaiian or Pacific Islander	2	0.47%
Prefer not to answer	17	3.97%
Other	15	3.50%
Marital Status (*n* = 428)		
Single	176	41.12%
Married	220	51.40%
Divorced	26	6.07%
Widow/widower	6	1.40%
Number of Children (*n* = 428)		
0	171	39.95%
1	73	17.06%
2	112	26.17%
more than 2	72	16.82%
Level of Education (*n* = 428)		
Not finished high school	2	0.47%
Finished high school	15	3.50%
Some college	49	11.45%
Associates degree	31	7.24%
Bachelor’s degree	113	26.40%
Master’s degree	74	17.29%
Doctoral degree	144	33.64%
Primary Role at Work (*n* = 428)		
Physician	122	28.50%
Nurses	94	21.96%
Administrative/clerical staff	65	15.19%
Patient Care associates	27	6.31%
Social worker	20	4.67%
Other	100	23.36%
Location of Work (*n* = 672)		
Administrative offices	75	11.16%
Ambulatory care	135	20.09%
Emergency Department	130	19.35%
Medical and Surgical Inpatient Units	127	18.90%
Intensive Care Units	84	12.50%
Operating Rooms	31	4.61%
Other	90	13.39%
Residence Borough (*n* = 428)		
The Bronx	150	35.05%
Manhattan	82	19.16%
Queens	49	11.45%
Brooklyn	19	4.44%
Staten Island	1	0.23%
Long Island NY	14	3.27%
Connecticut	2	0.47%
New Jersey	41	9.58%
Prefer not to answer	2	0.47%
Other	68	15.89%

**Table 2:** Fit statistics for each latent variable and full measurement model.

	RMSEA	CFI	TLI	SRMR	Chi-Square Test		
					χ^2^	df	*p*-Value
Full Measurement Model	0.067	0.913	0.882	0.076	2086.86	120	<0.001
Latent Variable and Associated Items (Factor Loading Indicated ^a^)							
Personal Risk							
How much of a problem is COVID-19 in America? ^b^ (−0.56)							
Based on your own health, age and risk factors, what do you believe to be your risk level were you to get COVID-19? ^b^ (0.30)							
Based on your overall experience with COVID-19 in both a professional and personal capacity, how serious would you say COVID-19? ^b^ (0.67)							
Vaccine Attitudes							
I am current on the vaccinations recommended by my primary care physician.^c^ (0.39)							
How important is it for you to get the flu vaccine every year? ^b^ (0.66)							
Vaccines are important for the prevention of serious diseases.^c^ (0.89)							
My children are current on recommended vaccines (or, if I don’t have children, I would keep my children current on recommended vaccines).^c^ (0.62)							
Location of Manufacture							
Development of a vaccine outside of the United States would reduce my likelihood of being vaccinated. ^c^ (1.18)							
Development of a vaccine in China or Russia would reduce my likelihood of being vaccinated ^c^ (0.90)							
Development of a vaccine in the United Kingdom would reduce my likelihood of being vaccinated ^c^ (1.30)							
Testing Speed							
I worry that the rushed pace of testing for the new COVID-19 vaccine will fail to detect potential side effects or dangers ^c^ (0.60)							
I would accept a COVID-19 vaccine with full FDA approval but not one with an emergency use authorization ^c^ (0.51)							
I would NOT accept a COVID-19 vaccine until the completion of its clinical trial and publication of results in a peer-reviewed journal such as JAMA, NEJM or the Lancet.^c^ (0.81)							
Likelihood of getting the COVID-19 vaccine							
I am likely to get the COVID vaccine ^d^ (0.82)							
Other people around me being vaccinated against COVID-19 will be helpful in controlling the pandemic ^c^ (−0.85)							
A vaccine is important to end the COVID-19 pandemic ^c^ (−0.94)							

^a^ All factor loadings are standardized. ^b^ These items were on a 5-point Likert scale indicating level of intensity (for specific statements, see full survey in the Supplementary Materials). ^c^ These items were on a 5-point Likert scale indicating level of agreement (for specific statements, see full survey in the Supplementary Materials). ^d^ This is a summed composite of five yes/maybe/no statements concerning the respondent’s willingness to receive the vaccine under different conditions: within 30 days, within six months, if it had to be administered yearly, if it was effective at preventing disease in 75% of receivers, and if it was effective at preventing disease in 90% of receivers.

**Table 3:** Multiple Regression Results.

Parameter	*B*	*SE B*	95% Wald CI		Wald Chi-Square	*df*	*p*-Value
			LL	UL			
(Intercept)	3.17	0.104	2.97	3.38	924.28	1	<0.001
Vaccine Attitudes	−0.091	0.006	−0.103	−0.08	202.37	1	<0.001
Testing Speed	0.014	0.005	0.004	0.024	7.30	1	0.007
Personal Risk	−0.017	0.008	−0.034	−0.001	4.57	1	0.033
Location of Manufacture	−0.007	0.005	−0.017	0.003	1.95	1	0.163
Education	0.005	0.011	−0.016	0.025	.21	1	0.646
Political Ideology	0.013	0.014	−0.014	0.040	.92	1	0.338

## Data Availability

Supporting data is available upon request to the authors. The overall data set is not available for publication as this was not approved by the IRB.

## Supplementary Materials

The following are available online at https://www.mdpi.com/article/10.3390/vaccines9050516/s1, Table S1: Complete Survey.
