# Supplementary material for: Knowledge, Attitudes and Perceptions of COVID-19 Vaccination among Healthcare Workers of an Inner-City Hospital in New York
Source: Vaccines (Basel). 2021 May 17;9(5):516. doi: 10.3390/vaccines9050516 (PMC8156250; doi:10.3390/vaccines9050516)
Supplement: Supplementary file 1 [file vaccines-09-00516-s001.zip › vaccines-1200918-supplementary.pdf]

Part 2A

Thank you for taking this survey. This is to help us understand how you feel about a potential vaccine for COVID-19. Your answers will be made anonymous so that no one will know how you personally answered each question.

Age

- ○ 18-25
- ○ 26-35
- ○ 36-45
- ○ 46-55
- ○ 55-65
- ○ Older than 65

Gender

- ○ Male
- ○ Female
- ○ Non-binary/third gender
- ○ ____________ Prefer to self-describe
- ○ Prefer not to answer

Race

- ○ American Indian or Alaska Native
- ○ Asian
- ○ Black or African American
- ○ Hispanic or Latino
- ○ Native Hawaiian or Pacific Islander
- ○ White
- ○ ____________ Other
- ○ Prefer not to answer

Based on your own health, age and risk factors, what do you believe to be your risk level were you to get covid-19?

- Would likely be asymptomatic or have mild disease
- Could be ill without requiring hospitalization
- Could be hospitalized
- Could be intubated or require severe interventions
- Could die

Please think about the people in your life, both personal and professional. What was the most severe outcome of COVID-19 among someone you know?

- No disease or I do not know anyone who had COVID-19
- Mild illness
- Moderate or severe illness but not requiring hospitalization
- Hospitalization
- Intensive care
- Death

Thinking about the people in your life, which relationship best describes the person who had the most severe disease outcome? (If you know more than one person who had that outcome, please select the closest relationship)

- Myself
- Immediate family member
- Extended family member
- Close friend
- Distant friend or acquaintance
- Patient or someone I cared for professionally
- I do not know anyone who had COVID-19

Based on your overall experience with COVID-19 in both a professional and personal capacity, how serious would you say COVID-19 is?

- I did not know anyone who had COVID-19
- Not very serious, most people have no trouble
- Moderate infection, similar to flu
- Serious, life-threatening infection
- Deadly

part 1: Demographic information

These questions are helpful to the researchers in comparing similar groups of people and to help us understand the results of the research better. Although these may be personal questions, your name and specific information will not be associated with the survey. Please select the best answer for each question.

Marital Staus

- Single
- Married
- Divorced
- Widow/widower

Number of children

- 0
- 1
- 2
- More than 2

Level of education

- Have not finished high school
- Finished high school
- Some college
- Associates degree
- Bachelors degree
- Masters degree
- Doctoral degree (e.g., MD,DDS,PhD)

Which option best describes your primary role at work?

- Physician
- Nurse Practicioner
- Physician Assistant
- Nurse
- Medical Student
- Patient Care Associate
- Maintenance staff
- Environmental Services
- Administrative support staff
- Physiotherapy, occupational therapy, speech and swallow
- Social worker
- Hospital police
- Dietary services/nutritionist
- Hospital administration
- ________ Other (please specify)

Please select the option that best describes your philosophical ideology

- ○ Very conservative
- ○ Somewhat conservative
- ○ Neither conservative nor liberal
- ○ Somewhat liberal
- ○ Very liberal

In which of the following settings do you regularly work, interact with patients and other staff members (select all that apply):

- ☐ Administrative offices
- ☐ Ambulatory Care
- ☐ Emergency department
- ☐ Med/Surg units
- ☐ Intensive care units
- ☐ Operating/procedure rooms
- ☐ ________ Other (please specify)

Part 2B

How has your experience with the COVID-19 global pandemic affected your opinion on vaccinations in general (not including a potential COVID-19 vaccine)?

- ○ I am much more likely to vaccinate myself/my children
- ○ I am more likely to vaccinate myself/my children
- ○ My opinion on vaccinations has not changed
- ○ I am less likely to vaccinate myself/my children
- ○ I am much less likely to vaccinate myself/my children

Why did the pandemic not change your opinion about vaccinations in general?

- I am already strongly in favor of vaccines, so it had little effect
- I am already strongly against vaccines, and it had little effect
- ________ Other

How have the COVID-19 lockdowns affected your ability to carry out your normal activities?

- Dramatic restrictions
- Moderate restrictions
- No restrictions
- Moderately easier
- Much easier

Which area do you live in?

- The Bronx
- Brooklyn
- Manhattan
- Queens
- Staten Island
- New Jersey
- Connecticut
- Long Island
- ________ Other

Which area do you work in?

- The Bronx
- Brooklyn
- Manhattan
- Queens
- Staten Island

Where do you believe you are most likely to contract Covid-19?

- In your community
- On the way to work
- At work

Do you live in an area where Covid-19 transmission is now increasing?

- Yes
- Unsure
- No

Part 2C

How closely do you follow news regarding COVID-19?

- Very closely
- Somewhat closely
- An average amount
- Not very closely
- Not at all

What is your primary source of information regarding COVID-19?

- Your primary doctor
- NYCHHC
- Other medical or scientific professionals (for example, an epidemiologist you follow on social media)
- CDC (Center for Disease Control and Prevention), WHO (World Health Organization) or NYC DOH
- Communications from professional organizations
- Public/political figures
- Scientific research or review studies
- Friends or Family
- News organizations: Print, TV, online, or mobile
- ________ Other (Please specify)

Part 2C (second half)

Please indicate whether the following statements are true or false:

Vaccines against pneumonia can protect against COVID-19

- True
- False

Certain antibiotics can prevent and/or treat COVID-19.

- True
- False

On average it takes 5–6 days from when someone is infected with COVID-19 for symptoms to show, however it can take up to 14 days.

- True
- False

Regularly rinsing your nose with saline can help prevent infection with COVID-19.

- True
- False

Once you contract COVID-19, the virus can never be eliminated from your body.

- True
- False

Symptoms of COVID-19 can include sore throat, diarrhea, and conjunctivitis (eye infection).

- True
- False

Most people who contract COVID-19 will recover from it.

- True
- False

Part 2D

I am current on the vaccinations recommended by my primary care physician.

- I am current on all
- I am current on most
- I am current on some
- I am not current on any
- I am uncertain

How important is it for you to get the flu vaccine every year?

- Very important
- Important
- Somewhat important
- Not very important
- Not at all important

Part 2E

In the next 30 days...

- I plan to vaccinate myself
- I will not vaccinate myself

Which of these statements most closely resembles your reason for choosing to not vaccinate yourself:

- I do not believe the vaccine is safe
- I do not believe the vaccine is effective
- I do not trust the source that encouraged me to get the vaccine
- I do not believe in any vaccines, and my reason is not any different for a new COVID-19 vaccine
- A source that I trust encouraged me to NOT get the vaccine
- I am indifferent to receiving the vaccine, but will probably end up not receiving it
- ________ Other

In the next 6 months...

- I plan to vaccinate myself
- I will not vaccinate myself

Which of these statements most closely resembles your reason for choosing to not vaccinate yourself:

- I do not believe the vaccine is safe
- I do not believe the vaccine is effective
- I do not trust the source that encouraged me to get the vaccine
- I do not believe in any vaccines, and my reason is not any different for a new COVID-19 vaccine
- A source that I trust encouraged me to NOT get the vaccine
- I am indifferent to receiving the vaccine, but will probably end up not receiving it
- ________ Other

If the COVID-19 vaccine would need to be administered yearly (similar to the flu shot), would you receive repeated vaccinations?

- Yes
- No

Which of the following most closely describes why you would be unlikely to receive a yearly vaccination administered at work?

- Limited time
- Concern with the vaccine itself
- Difficult to access Occupational Healthcare
- ______ Other

If the covid-19 vaccine were administered to you at the same time as the influenza vaccine (flu shot) would you be more likely to receive repeated vaccinations?

- Yes
- No

I am less likely to receive the vaccine because it has to be administered in two separate doses.

- True
- False

Which of the following most closely describes why you would be unlikely to receive a vaccination requiring multiple doses?

- Limited time
- Concern with the vaccine itself
- Difficult to access Occupational Healthcare
- ______ Other

If a vaccine for COVID-19 was made available and you were told it would protect half of the people who received it, how likely would you be to be vaccinated?

- Unlikely
- Unsure
- Likely

If a vaccine for COVID-19 was made available and you were told it would protect 3/4 of those who received it, how likely would you be to be vaccinated?

- Unlikely
- Unsure
- Likely

If a vaccine for COVID-19 was made available and you were told it would protect 90% of those who received it, how likely would you be to be vaccinated?

- Unlikely
- Unsure
- Likely

Part 2F

Vaccines are important for the prevention of serious diseases.

- Strongly agree
- Agree
- Uncertain
- Disagree
- Strongly disagree

The side effects of most vaccines are greater than the benefits

- Strongly agree
- Agree
- Uncertain
- Disagree
- Strongly disagree

I worry that the rushed pace of testing for a new COVID-19 vaccine will fail to detect potential side effects or dangers.

- Strongly agree
- Agree
- Uncertain
- Disagree
- Strongly disagree

My children are current on which recommended vaccines (or, if I don't have children, I would keep my children current on which recommended vaccines)?

- All recommended vaccines
- Most recommended vaccines
- Some recommended vaccines
- Uncertain
- My children are not/would not be vaccinated

Other people around me being vaccinated against COVID-19 will be helpful in controlling the pandemic

- Strongly agree
- Somewhat agree
- Neither agree nor disagree
- Somewhat disagree
- Strongly disagree

A vaccine is important to end the COVID-19 pandemic

- Strongly agree
- Somewhat agree
- Neither agree nor disagree
- Somewhat disagree
- Strongly disagree

Part 2G

A warning from the vaccine manufacturer concerning which of these side effects would prevent you from getting vaccinated? (select all that apply)

- [ ] Soreness at the site of injection
- [ ] Generalized muscle pain
- [ ] Fever
- [ ] Allergic reaction - mild or moderate (rash, temporary nausea, itchy skin)
- [ ] Allergic reaction - severe or life-threatening (difficulty breathing, choking sensation, dizziness, loss of consciousness)
- [ ] Temporary or permanent paralysis
- [ ] Autism spectrum disorder
- [ ] None of the above
- [ ] ________ Other

What frequency of serious side effects would stop you getting vaccinated against Covid-19?

- More than 1 in 10 people who were vaccinated
- More than 1 in 100 people who were vaccinated
- More than 1 in 1000 people who were vaccinated
- More than 1 in 10,000 people who were vaccinated
- More than 1 in 100,000 people who were vaccinated
- More than 1 in 1,000,000 people who were vaccinated
- No frequency of side effects would stop me from getting vaccinated

Please rank how much you agree with the following statements

	Disagree	Neither agree nor disagree	Agree
Receiving a COVID-19 vaccine at the same time as regularly scheduled vaccines would make me more likely to accept it.	○	○	○
I am worried that the vaccine itself will give me COVID-19.	○	○	○
I would rather build immunity by exposure to an infected individual than receive the vaccine.	○	○	○
Not everyone who is eligible for the vaccine needs to receive it because herd immunity is sufficient to protect everyone.	○	○	○
I am worried about the cost of a COVID-19 vaccine.	○	○	○
I am worried about side effects of the vaccine for myself.	○	○	○
The side effects of the vaccine are likely to be worse than COVID-19 itself	○	○	○

I trust the following measures to protect myself and others from COVID-19 **in public** if used correctly (select all that apply):

- ☐ Wearing a mask while in public
- ☐ Other people wearing masks
- ☐ Social Distancing
- ☐ Public sanitation measures like frequent handwashing and disinfecting surfaces
- ☐ None of the above

I currently take the following measures to protect myself and others from COVID-19 **in public** (select all that apply):

- ☐ Wearing a mask while in public
- ☐ Social distancing
- ☐ Public sanitation measures like frequent handwashing and disinfecting surfaces
- ☐ None of the above

How much of a problem is COVID-19 in America?

- ○ Not a problem at all
- ○ Insignificant compared to other problems
- ○ Somewhat of a problem
- ○ A severe problem, more important than most other issues
- ○ The most important problem facing America right now

I would accept a COVID-19 vaccine with full FDA approval but not one with an emergency use authorization.

- ○ Strongly agree
- ○ Somewhat agree
- ○ Neither agree nor disagree
- ○ Somewhat disagree
- ○ Strongly disagree

I would NOT accept a COVID-19 vaccine until the completion of its clinical trial and publication of a results in a peer-reviewed journal such as JAMA, NEJM or the Lancet.

- Strongly agree
- Somewhat agree
- Neither agree nor disagree
- Somewhat disagree
- Strongly disagree

Development of a vaccine outside of the United States would reduce my likelihood of being vaccinated

- Strongly agree
- Somewhat agree
- Neither agree nor disagree
- Somewhat disagree
- Strongly disagree

Development of a vaccine in China or Russia would reduce my likelihood of being vaccinated

- Strongly agree
- Somewhat agree
- Neither agree nor disagree
- Somewhat disagree
- Strongly disagree

Development of a vaccine in the United Kingdom would reduce my likelihood of being vaccinated

- Strongly agree
- Somewhat agree
- Neither agree nor disagree
- Somewhat disagree
- Strongly disagree

Block 10

Glucocorticoids (steroids) are recommended in the treatment of moderate to severe covid-19 infection

- True
- False
- Unsure

Systemic anticoagulation (blood thinner) is recommended in the treatment of covid-19 infection with venous thromboembolism (blood clots)

- True
- False
- Unsure

Remdesivir (antiviral) is recommended in the treatment of moderate to severe covid-19 infection

- True
- False
- Unsure

Hydroxychloroquine is recommended in the treatment of moderate to severe covid-19 infection

- True
- False
- Unsure

Block 11

Please indicate how confident you are that each of the following measures will protect you and your colleagues from contracting COVID-19 **at work**

	Not effective	Somewhat effective	Highly effective
Correct use of N95 Masks	○	○	○
Correct use of protective eyewear	○	○	○
Correct use of gloves	○	○	○
Correct use of aprons/gowns	○	○	○
Patient isolation	○	○	○
Negative pressure rooms	○	○	○
Rapid testing for patients upon admission	○	○	○
Mobile daily staff screening	○	○	○
Cleaning and disinfecting surfaces and rooms	○	○	○

I currently take the following measures to protect myself and others **at work** (select all that apply)

- ☐ Correct N95 mask use
- ☐ Correct use of protective eyewear
- ☐ Correct use of gloves
- ☐ Correct use of aprons/gowns
- ☐ Frequent testing, more than once per month

Block 12

Which of the following best describes your history of Covid-19 antibody testing?

- ○ I have never tested positive for antibodies
- ○ I have tested positive for antibodies once and have never been tested again
- ○ I have tested positive for antibodies multiple times with a stable or increasing titer
- ○ I have tested positive for antibodies multiple times but my titer is decreasing
- ○ I have tested positive for antibodies at least once but I now test negative

Please indicate how much you agree or disagree with the following statement: presence of antibodies against covid-19 confers sterile immunity, meaning you cannot get sick yourself or spread the virus to anybody else

- ○ Strongly agree
- ○ Somewhat agree
- ○ Neither agree nor disagree
- ○ Somewhat disagree
- ○ Strongly disagree

Please indicate how much you agree or disagree with the following statement: Once you no longer test positive for antibodies against covid-19, you are as likely to catch and spread the virus as someone who has never been infected

- Strongly agree
- Somewhat agree
- Neither agree nor disagree
- Somewhat disagree
- Strongly disagree

Following vaccination against COVID-19, the vaccinated person:

	Disagree	Unsure	Agree
Need no longer wear a mask or face covering in public	○	○	○
Need no longer wear N95, eye protection, gown/apron or gloves at work	○	○	○
Need no longer wash their hands between seeing patients	○	○	○
Need no longer adhere to social distancing measures in public	○	○	○
Need no longer adhere to social distancing measures at work	○	○	○

Powered by Qualtrics
